# Brief Diagnostic Criteria for Temporomandibular Disorders (Tmd): Enhancing Sensitivity in Diagnosing Headache Attributed to Tmd (A Multi‐Centre Study)

**DOI:** 10.1111/joor.70166

**Published:** 2026-02-14

**Authors:** Nykänen Laura, Kämppi Antti, Durham Justin, Eli Ilana, Freidman‐Rubin Pessia, Keren Lihi, Näpänkangas Ritva, Shalev Antshel Tamar, Tanner Johanna, Teerijoki‐Oksa Tuija, Winocur Ephraim, Sipilä Kirsi, Emodi‐Perlman Alona

**Affiliations:** ^1^ Clinic of Oral and Maxillofacial Diseases, Head and Neck Center Helsinki University Central Hospital Helsinki Finland; ^2^ Department of Oral and Maxillofacial Diseases, Faculty of Medicine University of Helsinki Helsinki Finland; ^3^ School of Dental Sciences, Newcastle University, Framlington Place. Newcastle. NE2 4BW. United Kingdom. Newcastle Hospitals' NHS Foundation Trust Newcastle‐Upon‐Tyne UK; ^4^ Department of Oral Rehabilitation, the Maurice and Gabriela Goldschleger School of Dental Medicine, Faculty of Medicine and Health Sciences Tel‐Aviv University Tel‐Aviv Israel; ^5^ Research Unit of Population Health, Faculty of Medicine, University of Oulu, Oulu, Finland, and Medical Research Center Oulu Oulu University Hospital and University of Oulu Oulu Finland; ^6^ Department of Oral and Maxillofacial Diseases Turku University Hospital Turku Finland; ^7^ Department of Oral and Maxillofacial Surgery, Institute of Dentistry University of Turku Turku Finland

**Keywords:** DC/TMDbDC, headache, temporomandibular, temporomandibular disorders, TMD

## Abstract

**Background:**

The brief Diagnostic Criteria for Temporomandibular Disorders (bDC/TMD) was developed to simplify the original DC/TMD for wider clinical use. While its diagnostic accuracy for most painful TMDs is acceptable, the sensitivity for headache attributed to TMD (HaTMD) was reported to be poor.

**Objectives:**

To improve the diagnostic sensitivity of HaTMD within the bDC/TMD framework by reintroducing selected examination items from the original DC/TMD protocol.

**Methods:**

This retrospective multicentre study used data from Finland and Israel. The cohort included 114 individuals previously diagnosed with HaTMD according to the DC/TMD and with myalgia and/or arthralgia diagnoses in both DC/TMD and bDC/TMD. Four examination items excluded from the bDC/TMD—E1b (headache location in temple), E4c (familiar headache on assisted opening), E5a/b (lateral movements) and E5c (protrusive movements)—were reintroduced individually. Four calibrated examiners reassessed each modified dataset. Inter‐examiner reliability (Cohen's kappa) and diagnostic sensitivity were calculated using DC/TMD as the gold standard.

**Results:**

Inter‐examiner reliability for HaTMD diagnosis was almost perfect (κ = 0.81–1.00) across all items. Sensitivity improved markedly from the previously reported 0.16–0.38 to 0.82 (E1b)–0.90 (E5c). Item E1b (temple headache confirmation) was present in 95% of Finnish and 86% of Israeli cases, identifying it as the most representative finding.

**Conclusion:**

Reintroducing item E1b into the bDC/TMD examination substantially increases the diagnostic sensitivity for HaTMD while maintaining brevity. Refinement of the painful TMD diagnostic decision tree and prospective validation of the modified bDC/TMD are recommended to ensure reliability and clinical applicability.

## Graphical abstract

This multicentre retrospective study evaluated reintroducing selected DC/TMD examination items into the bDC/TMD to improve diagnosis of headache attributed to TMD. Reintroducing temple headache confirmation (E1b) markedly increased diagnostic sensitivity while maintaining high inter‐examiner reliability, supporting refinement and validation of the modified protocol.

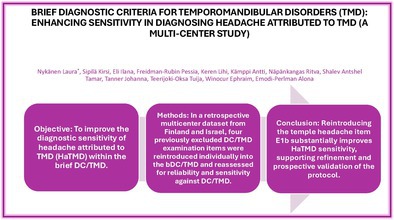

## 1 Introduction

Temporomandibular disorders (TMD) encompass a range of conditions that can affect some or all the masticatory muscles, temporomandibular joints, and related structures. Symptoms can vary, with their intensity naturally fluctuating over time [1, 2]. A recent meta‐analysis reported a significant global prevalence of 34% [3].

Reliable and valid diagnostic criteria for TMD are essential for both clinical practice and research. Standardised instruments include the Research Diagnostic Criteria (RDC) for TMD [4] and the Diagnostic Criteria (DC) for TMD, introduced in 2014 by the International Network for Orofacial Pain & Related Disorders Methodology (INfORM) [5]. The DC/TMD was validated through a large‐scale population study [6] and aligns with the biopsychosocial model of TMD [7]. It is recognised as the gold‐standard instrument for TMD diagnosis [5, 8], but its adoption in general clinical settings remains limited [9, 10]. One of the challenges associated with its implementation is the time‐intensive nature of its physical examination, requiring extensive examiner training. Its psychosocial assessment is also challenging to integrate into routine clinical workflows [11].

To address these challenges, INfORM initiated the development of a streamlined version of the DC/TMD, the brief Diagnostic Criteria (bDC) for TMD, an abbreviated version of the original DC/TMD [12]. The initial publication of the bDC/TMD highlighted the need for further psychometric evaluation and field testing to refine the instrument and assess its feasibility [12].

A recent multi‐centre study examined the sensitivity and specificity of Axis I clinical diagnoses of the bDC/TMD against the gold standard DC/TMD [13]. The bDC/TMD demonstrated good sensitivity for most painful TMD conditions, except for headache attributed to TMD (HaTMD), for which the sensitivity was poor (ranging 0.16–0.38 among examiners). This represents a significant decrease from the original DC/TMD, in which sensitivity for HaTMD was markedly higher (0.896) [5].

HaTMD is a secondary headache disorder that frequently is comorbid with primary headache conditions such as tension‐type headache and migraine [14, 15]. Symptoms and clinical findings of HaTMD and primary headaches can mimic each other, and differential diagnostics are challenging [15, 16]. Unlike primary headaches that are mainly of neurological origin, TMD‐associated headaches are secondary manifestations of myalgia and/or arthralgia TMD, located in the temple/temporalis region, characteristically exacerbated by jaw movement and masticatory function [5, 16]. Accurate differentiation between headache types is clinically essential as therapeutic interventions vary considerably, and that is why it is essential to also include in the bDC/TMD with reasonably sensitive diagnostic accuracy. While primary headaches fall mainly under the responsibility of neurologists, headaches attributed to TMD can be managed by dental professionals. TMD‐focused treatments are beneficial for TMD headaches but may prove ineffective for primary headache conditions [17]. The frequent comorbidity necessitates thorough clinical evaluation to determine the predominant condition and prevent misdiagnosis of a concurrent pathology requiring specific management approaches [17].

According to the DC/TMD, diagnosis of HaTMD can be established when five criteria are present simultaneously: (i) patient has muscle pain (myalgia) or joint pain (arthralgia) that meets the DC/TMD diagnostic standards; (ii) patient‐reported temple headache which is modified by jaw function; (iii) clinical confirmation of headache location in the temple through clinical examination; (iv) familiar headache while palpating the temporalis muscle or performing jaw movements; and (v) headache is not better accounted for by other headache diagnoses [5].

This study aimed to enhance the diagnostic sensitivity of haTMD of the bDC/TMD tool by incrementally reintroducing selected items from the DC/TMD tool into the brief DC/TMD framework.

## 2 Material and Methods

### 2.1 Study Protocol

This is the second phase of a larger retrospective study based on patient records aimed at the evaluation of bDC/TMD diagnoses, for which the initial results have been published previously [13]. The study encompassed four centres in Finland (Clinics for Oral and Maxillofacial Diseases at Helsinki, Oulu, Kuopio and Turku University Hospitals) and one centre in Israel (students' TMD dental clinic at the Goldschleger School of Dental Medicine, Tel Aviv University—TAU).

In the first phase, files of 334 subjects (Finland and Israel), who were initially diagnosed according to the DC/TMD protocol, were reduced to include only information included in the bDC/TMD protocol and re‐evaluated for TMD diagnoses. Information regarding the sensitivity and specificity of the bDC/TMD diagnoses (as compared to the gold standard DC/TMD) can be found in Nykänen et al. [13].

**FIGURE 1:**
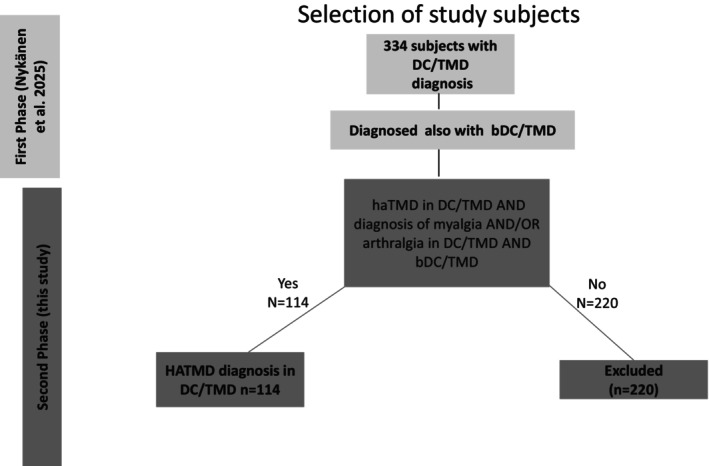
Selection of subjects for the study.

In the second phase (present study), the study population included a sub‐cohort of individuals who received a diagnosis of HaTMD according to the original DC/TMD protocol. As myalgia or arthralgia is a prerequisite for HaTMD diagnosis [5], only individuals with a diagnosis of myalgia and/or arthralgia both in the DC/TMD and the bDC/TMD protocols were included in the group. Concludingly, all subjects in the present study had an initial diagnosis of HaTMD in the original, gold standard DC/TMD protocol and a diagnosis of myalgia or myalgia and arthralgia in both instruments (DC/TMD and bDC/TMD) (Figure 1). In this study population, no individual had a diagnosis of arthralgia only and HaTMD. In the Finnish data, four individuals and in the Israeli data, five individuals had a haTMD diagnosis but item E1b (Examiner confirmation of headache location) was negative. They were included in the study population, as the original DC/TMD diagnosis was made by experienced, calibrated examiners, based on familiar headache referred from other masticatory structures upon palpation.

To enhance diagnostic sensitivity of HaTMD through the bDC/TMD protocol, items from the original DC/TMD, which have been excluded from the bDC/TMD, were re‐introduced to the database as follows:

1. All items referring to the diagnosis of HaTMD in the DC/TMD protocol were listed (Table 1)

**TABLE 1:** Outcomes and items necessary for HaTMD diagnosis: Presence/absence in the bDC/TMD protocol and decision for re‐introduction to the bDC/TMD protocol.

Outcome or item^a^, ^b^ (number/description)	DC/TMD	bDC/TMD
Diagnostic outcome of executed criteria of myalgia or arthralgia	Yes	No
SQ5: Headache of any type in temporal region	Yes	Yes
SQ7: Headache modified by jaw movement, function or parafunction	Yes	Yes
E1b: Examiner confirmation of headache in temporalis area	Yes	No
E4b: Familiar headache from unassisted opening	Yes	Yes
E4c: Familiar headache from assisted opening	Yes	No
E5a or E5b: Familiar headache from lateral excursive movements	Yes	No
E5c: Familiar headache from protrusive excursive movements	Yes	No
E9: Familiar headache from temporalis muscle palpation	Yes	Yes

^a^ SQx – numbered item is a question in the self‐report Symptom Questionnaire.

^b^ Ex—Numbered item is an examination item in the examination protocol.

**FIGURE 2:**
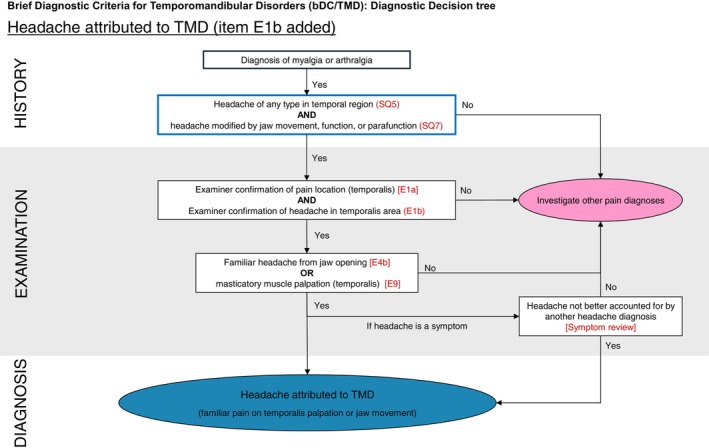
Modified Brief Diagnostic Decision Tree for Headache Attributed to Temporomandibular Disorders, with Clinical Examination item E1b (Examiner confirmation of headache in the temporalis area) added.

2 Each of the outcomes and items (see Table 1, column 4) was re‐introduced to the database, one item at a time. This way, four new databases were formed with the raw data for existing items for bDC/TMD haTMD diagnosis and reintroduced items as follows: (i) database including item E1b; (ii) database including item E4c; (iii) database including item E5a or b; and (iv) database including item E5c.
3 A modified Diagnostic Decision Tree for HaTMD was developed, following the algorithm from the DC/TMD Diagnostic Decision Tree for Painful TMD conditions. The reintroduced diagnostic outcome was integrated into the tree structure according to the algorithm used in the original bDC/TMD Diagnostic Tree for Painful TMD and Headache. (Figure 2). In the Finnish data, four individuals and in the Israeli data five individuals had a haTMD diagnosis but item E1b was negative. They were included in the study population, as the diagnosis was made by experienced, calibrated clinicians, based on a referred familiar headache from item E9 from other masticatory structures.

Based on the available information, four Level III calibrated examiners, who participated in the 1st phase of the study (two Finnish for the Finnish data and two Israeli for the Israeli data), gave their decision regarding the presence or absence of HaTMD (yes/no) to all subjects in all four databases with the re‐introduced items. To avoid recall bias, the data was anonymised and the order of subjects in the databases was randomised by computer for each re‐introduced item when the examiner switched to the next database.

### 2.2 Statistical Analysis

Inter‐rater reliability was evaluated using Cohen's kappa, separately for each group of examiners (Finnish examiners, *n* = 2; Israeli examiners, *n* = 2).

Sensitivity of the HaTMD diagnosis was calculated separately for each of the added items. Only subjects with absolute positive or negative agreement between the two examiners in Finland and in Israel were considered. Sensitivity was calculated as compared to the original DC/TMD diagnosis which served as the gold standard. As the aim of the original bDC/TMD tool was to keep the instrument short and simple [12] an attempt to further increase sensitivity by calculation of combinations of items was not performed. Specificity was not calculated, as no gold standard true negatives of HaTMD were included. The study received a permit from the Ethics Committee of the Hospital District of Southwest Finland (74/1082/2015), the Helsinki University Central Hospital Head and Neck Center (permit no. HUS/53/2023) in Finland, and from the Tel Aviv University Ethical Committee (permit no 0008783‐1).

## 3 Results

The study included data from a total of 114 subjects: 79 in Finland (11.4% males) and 35 in Israel (28.6% males).

Inter‐examiner reliability of HaTMD diagnosis, per each re‐introduced item, was almost perfect (Table 2).

**TABLE 2:** Sensitivity of bDC/TMD for HaTMD and inter‐examiner reliability of re‐introduced items.

Re‐introduced item	Finland (*n* = 79)			Israel (*n* = 35)		
	Sensitivity	Cohen's kappa^a^	Positive findings (*n*/%)	Sensitivity	Cohen's' kappa^a^	Positive findings (*n*/%)
E1b	0.82	1,00	75/94.9	0.886	1.00	30/85.7
E4c	0.859	0,95	14/17.7	0.824	0.96	2/5.71
E5a or b	0.873	1,00	5/6.33	0.824	0.96	1/2.86
E5c	0.897	0,93	4/5.06	0.826	1.00	1/2.86

^a^ inter‐examiner reliability, Cohen's kappa: 0.81–1.00 almost perfect.

Sensitivity of the HaTMD diagnosis was calculated against the gold standard DC/TMD instrument, as published by Shiffman et al. [5] Sensitivity of the HaTMD diagnosis, per added item, ranged from 0.82 (item E1b) to 0.897 (item E5c), which shows excellent sensitivity for each one of the re‐introduced items (Table 2). Among the re‐introduced items, item E1b (headache located in the temple area) was the most common in both populations, present in almost 95% of the cases diagnosed with HaTMD (Table 2).

## 4 Discussion

The team's previous study demonstrated the poor sensitivity (0.16–0.38) of HaTMD diagnosis of the bDC/TMD [13]. The present study aimed at enhancing the bDC/TMD diagnostic sensitivity by reintroducing items, which had been initially removed from the instrument. Results show that re‐introduction of any of the items referring to HaTMD, which have been removed from the original DC/TMD protocol, markedly improves the sensitivity of the bDC/TMD protocol as far as the diagnosis of HaTMD is concerned.

Notably, headache located in the temple area (item E1b) was present in nearly all Finnish and Israeli subjects, suggesting it is the most representative clinical finding for HaTMD. Since examining the temple area during clinical assessment requires only a brief time investment, it would be justified and advisable to reintegrate this component into the bDC/TMD clinical examination protocol. Such modification to the bDC/TMD would maintain the instrument's originally intended brevity and simplicity while significantly enhancing its sensitivity for diagnosing HaTMD.

Both inter‐examiner reliability and sensitivity showed significant improvement compared to the previous study, with better consistency between examiners and populations. Our previous study displayed marked sensitivity differences between the Finnish and Israeli data, which were then interpreted to mainly stem from differences of the study populations (primary vs. tertiary) [13]. This study presents very similar sensitivity in all items examined; however, this study only measures item performance of haTMD clinical examination items, whereas our previous study measures entire diagnosis constructs. Sensitivity differences are thus not comparable. Our previous study highlighted the interpretive challenges inherent in the Diagnostic Decision Tree for Painful TMD [13]. The first decision box originally proposed in the bDC/TMD was subject to multiple interpretations. To address this gap, a modified diagnostic tree that incorporates the additional examination item for HaTMD diagnosis was created (Figure 1). The approach maintained strict adherence to the brief decision tree algorithm while strategically integrating the new examination component into the appropriate decision point, following the structural framework of the original DC/TMD Painful TMD Diagnostic Decision Tree. Furthermore, myalgia or arthralgia diagnosis was incorporated as a mandatory prerequisite for initiating the HaTMD diagnostic algorithm, consistent with the original DC/TMD diagnostic decision tree methodology.

When decision trees use strict dichotomous criteria, results become more harmonious, as compared to the previous study's poor results for HaTMD interrater reliability and sensitivity. Based on these findings, we propose two modifications to the Painful TMD Diagnostic Decision Tree of the original bDC/TMD: (i) implementing more dichotomous, clear‐cut decision points in each diagnostic box regarding anatomical locations leading to the diagnosis to reduce interpretive ambiguity; and (ii) restructuring the HaTMD diagnosis pathway to mirror the original DC/TMD approach by beginning with a myalgia or arthralgia diagnosis determination (‘Diagnosis of myalgia YES/NO’) before proceeding through the diagnostic algorithm outlined in Figure 1. The diagnostic decision‐making would then require more time, but as headache differential diagnostics is crucial for correct management or referral of the patient, we consider the additional time investment justified. Future studies could compare execution times of the original and brief DC/TMD's of different professional groups (students, general dentists, specialists), but this requires for the brief instrument to have reached its final form.

Both populations consistently identified item E1b (familiar headache location on the temple) as the most representative HaTMD finding. The study revealed an important finding for clinical screening: patients in the tertiary clinic (Finland) showed minimal clinical signs during assisted jaw opening and/or extrusive movements (items E4C, E5). This suggests that haTMD can be accurately diagnosed in chronic TMD patients without requiring the more complex examination procedures. Since HaTMD can be identified without specialised jaw manipulation techniques, headache specialists from non‐dental backgrounds, such as neurologists or general practitioners, could potentially screen for this condition when evaluating patients with other types of headaches. The simplified diagnostic approach removes technical barriers that might otherwise require specialised dental or TMD training and calibration. The diagnosis of haTMD requires high sensitivity and specificity in the general dental setting, as for example migraine‐type headaches are often concurrent in subjects with HaTMD are both mediated through the trigeminal system, but require different diagnostics and management [17, 18]. A traffic‐light approach has been suggested, where the red and yellow light phases are differential screenings of primary headaches, and the green light phase is actual HaTMD diagnostics [14]. The bDC/TMD followed this approach, namely by including red flag signs of headache requiring immediate referral and a migraine screening tool, but showed poor sensitivity for HaTMD, thus being short of the essential diagnostic ‘green light’ phase. The suggestion to add item E1b and refine the Painful TMD's Diagnostic Decision Tree can resolve this shortcoming of the original bDC/TMD instrument. Re‐introducing item E1b will result in the following items being part of the bDC/TMD HaTMD diagnosis: (i) patient has muscle pain (myalgia) or joint pain (arthralgia) that meets the DC/TMD diagnostic standards; (ii) patient‐reported temple pain which is modified by functional movements (SQ5 and 7); (iii) clinical confirmation of temple involvement through physical examination (E1a and b); (iv) headache changes in response to jaw function or movement (E4) or familiar pain response while palpating the area or performing jaw movement tests (E9).

The retrospective design, using existing datasets rather than prospective evaluation of live subjects, is not optimal and can be considered a limitation of the study. Preferably, participants would have been clinically assessed twice by two independent examiners: once with the complete DC/TMD protocol and again with the bDC/TMD to provide direct comparative validation. Cross‐evaluation between Finnish and Israeli data and examiners was not possible due to data sharing restrictions outside the European Union of the study permit in Finland. Additionally, sample size was relatively small due to restricting analysis to subjects who have already been diagnosed with HaTMD according to the DC/TMD criteria. The missing of subjects with arthralgia only and HaTMD in the gold standard DC/TMD in this dataset doesn't allow for conclusions about whether arthralgia must be included as a prerequisite for HaTMD in the newly proposed bDC/TMD Diagnostic Decision Tree for HaTMD. A previous study by Exposto et al. suggests temporal myalgia and HaTMD to be diagnosed as the same entity [19]. However, the present study's methodology or results do not provide enough support to recommend the omittance of arthralgia as a prerequisite for diagnosis of haTMD in the bDC/TMD. The methodology of the present study only allows the measurement of the performance of separate items in the clinical examination, not the criteria performance of haTMD. Future studies should employ larger DC/TMD databases and, more importantly, conduct prospective, blinded clinical studies in diverse population samples, with asymptomatic individuals as well, to detect possible false positives, to re‐test and generalise the results of this study, and to further validate the psychometric properties of the bDC/TMD instrument.

The study's primary strength lies in its attempt to address a critical gap in TMD diagnostics. To the best of our knowledge, this is the first investigation to systematically analyse and provide evidence‐based solutions for the diagnostic shortcomings of the bDC/TMD instrument that have been identified in the initial reliability study [13]. The rigorous methodology, combined with data from two different healthcare settings (primary and tertiary care), provides evidence for the instrument's clinical utility. The study actively improves upon existing diagnostic tools, potentially expanding access to TMD screening across medical specialties that previously lacked practical diagnostic options.

## 5 Conclusions

Re‐introduction of item E1b (confirmation of headache located in the temple area) to the clinical examination of the bDC/TMD protocol can substantially enhance the diagnostic sensitivity of haTMD. We recommend refining the painful TMD diagnostic decision tree by establishing a dedicated branch for HaTMD and mandating at least a myalgia diagnosis as the initial requirement. A prospectively collected clinical dataset could test the full reliability of the instrument after these suggested alterations. Following the finalisation of these improvements to the bDC/TMD instrument, publication of the complete tool as an appendix will be essential for advancing future psychometric research and facilitating widespread adoption in clinical practice.

## Author Contributions

**Nykänen Laura:** study design, analysis and interpretation of results, drafting and finalising the manuscript; **Sipilä Kirsi:** study design, data acquisition, conceptualization; **Eli Ilana:** study design, interpretation of results, conceptualization; **Durham Justin:** conceptualization, finalising the manuscript; **Freidman‐Rubin Pessia**, **Keren Lihi, Näpänkangas Ritva**, **Shalev Antshel Tamar**, **Tanner Johanna**, **Teerijoki‐Oksa Tuija**, **Winocur Ephraim:** data acquisition; **Kämppi Antti:** data analysis; **Emodi‐Perlman Alona:** study design, interpretation of results, conceptualization, finalising the manuscript. All authors have contributed intellectually to the manuscript by commenting and revising it.

## Funding

This work was supported by Head and Neck Center, Helsinki University Hospital.

## Ethics Statement

The study has received a permit from the Ethics Committee of the Hospital District of Southwest Finland (74/1082/2015), the Helsinki University Central Hospital Head and Neck Center (permit no. HUS/53/2023) in Finland, and the Tel Aviv University Ethical Committee (permit no 0008783–1).

## Conflicts of Interest

The authors declare no conflicts of interest.

## Data Availability

The data that support the findings of this study are available on request from the corresponding author. The data are not publicly available due to privacy or ethical restrictions.
